# A small caseid synapsid, *Arisierpeton simplex* gen. et sp. nov., from the early Permian of Oklahoma, with a discussion of synapsid diversity at the classic Richards Spur locality

**DOI:** 10.7717/peerj.6615

**Published:** 2019-04-11

**Authors:** Robert R. Reisz

**Affiliations:** International Centre of Future Science, Jilin University, Changchun, Jilin, China; Department of Biology, University of Toronto Mississauga, Mississauga, Ontario, Canada

**Keywords:** Early synapsid, Early permian period, Synapsid diversity, Herbivory, Dolese, Richards spur, Oklahoma

## Abstract

The fossil record of caseids, a clade of faunivorous to large herbivorous Permian synapsids, is unusual in having a poorly documented history. Although Kungurian caseids are common in the well-known continental deposits of North America, and the fossil record of the group extends into the middle Permian (Guadalupian), with the presence of the large caseid *Ennatosaurus* in the Mezen Basin faunal assemblage, only two other occurrences are known in older Permian age sediments. One is an undescribed caseid from the Bromacker Quarry in Germany, and the second is *Oromycter* from the lower Permian of Richards Spur, Oklahoma. The former is known from several articulated skeletons, but the latter is known only from a handful of skeletal elements, including elements of the snout and lower jaw, some phalanges, and a few vertebrae. Here the fragmentary tooth bearing elements and dorsal vertebrae of another small caseid from Richards Spur are described, with a discussion of its significance in the context of caseid evolution, and the continuously expanding faunal list and taxic diversity at this locality.

## Introduction

Caseids have a unique evolutionary history among early synapsids, both in terms of their anatomy and fossil record. Caseids belong to the most basal clade of Synapsida, the Caseasauria (Reisz & Fröbisch, 2014). All members of the clade are characterized by a number of cranial and postcranial characters (Reisz, 2005; LeBlanc & Reisz, 2014), including a disproportionately small cranium with a procumbent snout that overhangs the tooth row, as seen in both small taxa like *Casea* and *Euromycter* (Reisz et al., 2011), and except for the most basal taxon *Eocasea* (Reisz & Fröbisch, 2014), an expanded rib cage with massive ribs, best seen in *Ruthenosaurus* (Reisz et al., 2011), *Cotylorhynchus* (Reisz, 1986), and *Ennatosaurus* (Olson, 1968). Most interestingly, the dental and jaw modifications seen in caseids showcase the earliest known example of the evolution of terrestrial vertebrate herbivory without dental occlusion (Reisz, 2006). In this particular feature, caseids and their dentition differ from all other early herbivorous vertebrates in evolving teeth that are clearly used in cropping and cutting vegetation but with little oral processing, and no dental occlusion (Maddin, Sidor & Reisz, 2008). This is in strong contrast to the condition seen in other late Pennsylvanian and early Permian tetrapods (diadectids and amniotes), in which the most common method of oral processing is accomplished by tooth-to-tooth contact that results in dental wear of the upper and lower dentition (Reisz, 2006; LeBlanc et al., 2015; Modesto et al., 2015). Members of the Eothyrididae, the sister clade to caseids (Reisz, Godfrey & Scott, 2009; Brocklehurst et al., 2016), and the oldest known caseid *Eocasea martini,* from the late Pennsylvanian of Kansas, USA (Reisz & Fröbisch, 2014) are all small faunivorous forms with simple, conical, slightly recurved teeth, indicating that the dental specializations for a non-occluding herbivorous dentition evolved within Caseidae.

The fossil record of caseids is also unusual in that the early history of the clade is poorly documented. Although Kungurian caseids are common in the lowland, floodplain continental deposits of North America, only two other occurrences are known in older Permian age sediments. One is an undescribed caseid from the Bromacker Quarry in Germany (Eberth et al., 2000), and the second is *Oromycter* (Reisz, 2005) from the lower Permian of Richards Spur, Oklahoma. The former is known from several articulated skeletons, but the latter is known only from a handful of skeletal elements, including elements of the snout and lower jaw, some phalanges, and a few vertebrae. Both of these localities are considered to preserve upland vertebrate communities (Eberth et al., 2000) and are the only sources of caseid remains at this time, raising the possibility that the initial stages of caseid evolution occurred in the uplands (MacDougall et al., 2017). In particular, the Dolese Quarry near Richards Spur contains a series of karst fissures within Ordovician limestone. These fissures have been infilled by early Permian fossiliferous sediments, and are interpreted as an extensive, complex cave system. This indicates that the locality represents a unique depositional environment, distinct from the typical coeval lowland/deltaic regimes found throughout Laurasia, preserving an exceptionally rich, diverse fauna of fully terrestrial vertebrates.

The coeval fossil record of lowland/floodplain communities that are abundantly preserved in Laurasia are dominated by ophiacodontid, sphenacodontid, and edaphosaurid synapsids (Reisz, 1986), and caseids are absent. However, two other potential basal caseids have been recently redescribed from Europe, *Callibrachion gaudryi* from the early Permian of northeastern France, and *Datheosaurus macrourus* from the late Carboniferous of Poland (Spindler, Falconnet & Fröbisch, 2015). Although these are possibly basal caseids (Brocklehurst et al., 2016), and potentially important because they appear relatively early in the caseid fossil record, the poor preservation of their skulls and dentitions makes overall interpretations of their placement among caseids somewhat problematic.

Here I describe the remains of another caseid from Richards Spur, and discuss its significance in the context of caseid evolution (Romano, Brocklehurst & Fröbisch, 2017), and the continuously expanding faunal list and taxic diversity at this locality.

## Materials and Methods

The specimens described herein were prepared manually using a dissecting microscope, and were photographed using a Canon EOS 40 D camera with an EF 100 mm macro lens. The SEM images were taken with a JEOL JCM-5000 Neoscope Table Top Scanning electron microscope.

In contrast to the vast majority of dark brown or black colored fossil materials that have been derived from the Richards Spur locality, the fossils described here were cream-colored. These materials were collected towards the end of the 20th century, and the fauna preserved in this region of the cave system includes numerous disarticulated materials of the trematopid *Acheloma* (Sullivan & Reisz, 2000)*,* and the large captorhinid *Captorhinus magnus* (Kissel, Dilkes & Reisz, 2002). All materials described here were apparently in close proximity to each other, but their disarticulated condition prevents any clear associations.

The specimens belong to the Giuseppe Alberto Arisi collection in Isolabona (Italy), and is legally registered by a decree of the Minister per I Beni a la Attivita Culturali, April 19th, 2017, DPCR 022/17, following the disposition of Italian law No. 42, dated January 22, 2004. The Soprintendenza per i Beni Archeologici della Liguria recognized in a report enclosed in the decree, this collection is being studied by Professor Robert Reisz.

Under Italian law, these specimens are recognized as of public and scientific interest, and cannot be sold. Italian law requires that the Giuseppe Alberto Arisi Collection be available for study. When and if the current Arisi collection cannot be properly curated and maintained, the Italian government will expropriate the owners, and the specimens will be given to the regional natural history museum for curation and maintenance.

The electronic version of this article in Portable Document Format (PDF) will represent a published work according to the International Commission on Zoological Nomenclature (ICZN), and hence the new names contained in the electronic version are effectively published under that Code from the electronic edition alone. This published work and the nomenclatural acts it contains have been registered in ZooBank, the online registration system for the ICZN. The ZooBank LSIDs (Life Science Identifiers) can be resolved and the associated information viewed through any standard web browser by appending the LSID to the prefix  http://zoobank.org/. The LSID for this publication is: urn:lsid:zoobank.org:pub:B97895AC-87A0-407E-9761-026B71899524

The online version of this work is archived and available from the following digital repositories: PeerJ, PubMed Central and CLOCKSS.

*Arisierpeton*: urn:lsid:zoobank.org:act:42D63CEE-144E-4593-A448-3B7AEA2D8D69.

*Arisierpeton simplex*: urn:lsid:zoobank.org:act:AD7D8FF9-6269-4B1C-A4DD-6F42B969F167.

## Systematic Paleontology

**Table:** 

SYNAPSIDA Osborn, 1903
CASEASAURIA Williston, 1911
CASEIDAE Williston, 1911
*ARISIERPETON SIMPLEX* gen. et sp. nov.

**Figure 1:**
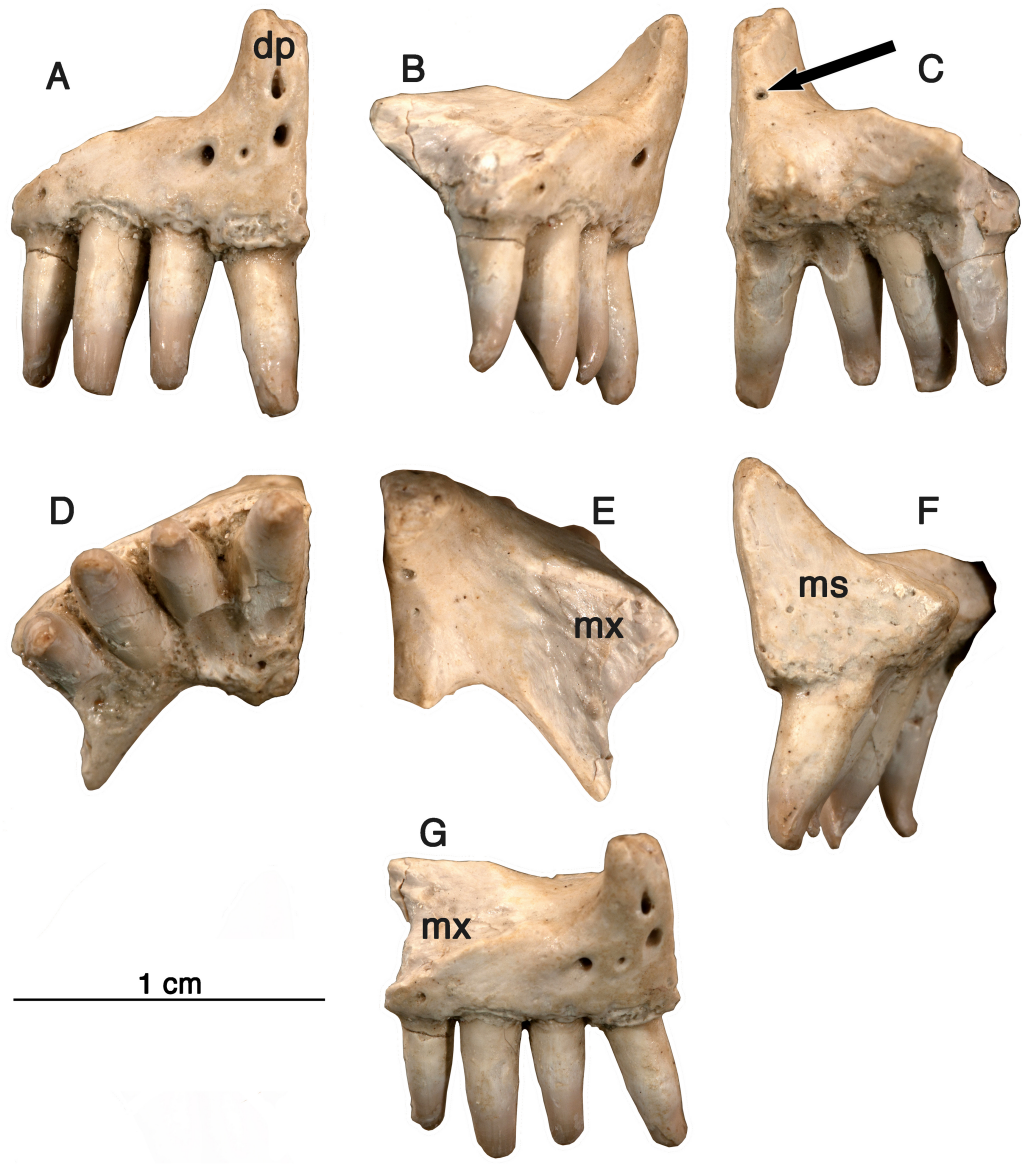
Holotype specimen, premaxilla. *Arisierpeton simplex,* holotype specimen GAA 00225-1. The right premaxilla in seven views (from left to right), (A) anterior surface showing premaxillary foramina; (B) partial labial or lateral view; (C) posterior view; (D) occlusal or ventral view; (E) dorsal view: (F) lingual view: (G) laterodorsal showing sutural surface with the maxilla. Arrow points to posterodorsal foramen.

**Holotype-**GAA 00225-1 (Fig. 1), a nearly complete right premaxilla.

**Figure 2:**
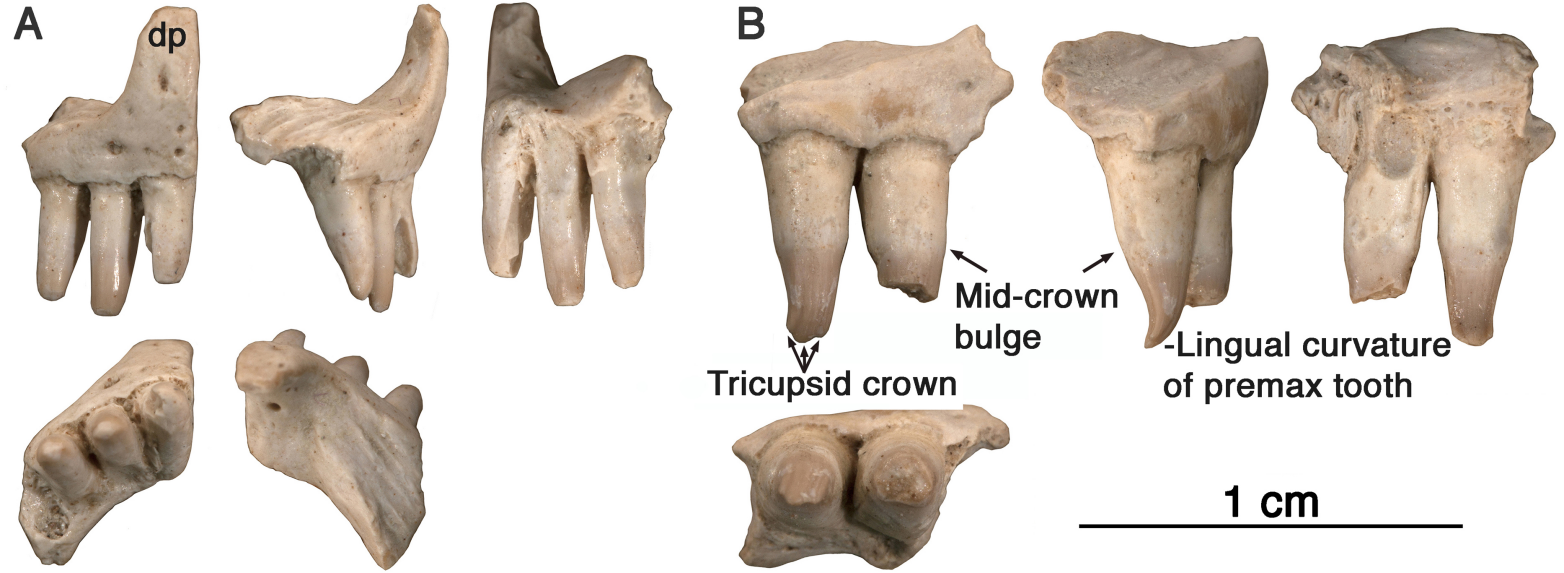
*Arisierpeton simplex,* premaxillae. (A) Images of GAA 00242 in anterior, labial, posterior, ventral and dorsal views; (B) images of GAA 00239 in labial, partial labial, posterior and ventral views.

**Figure 3:**
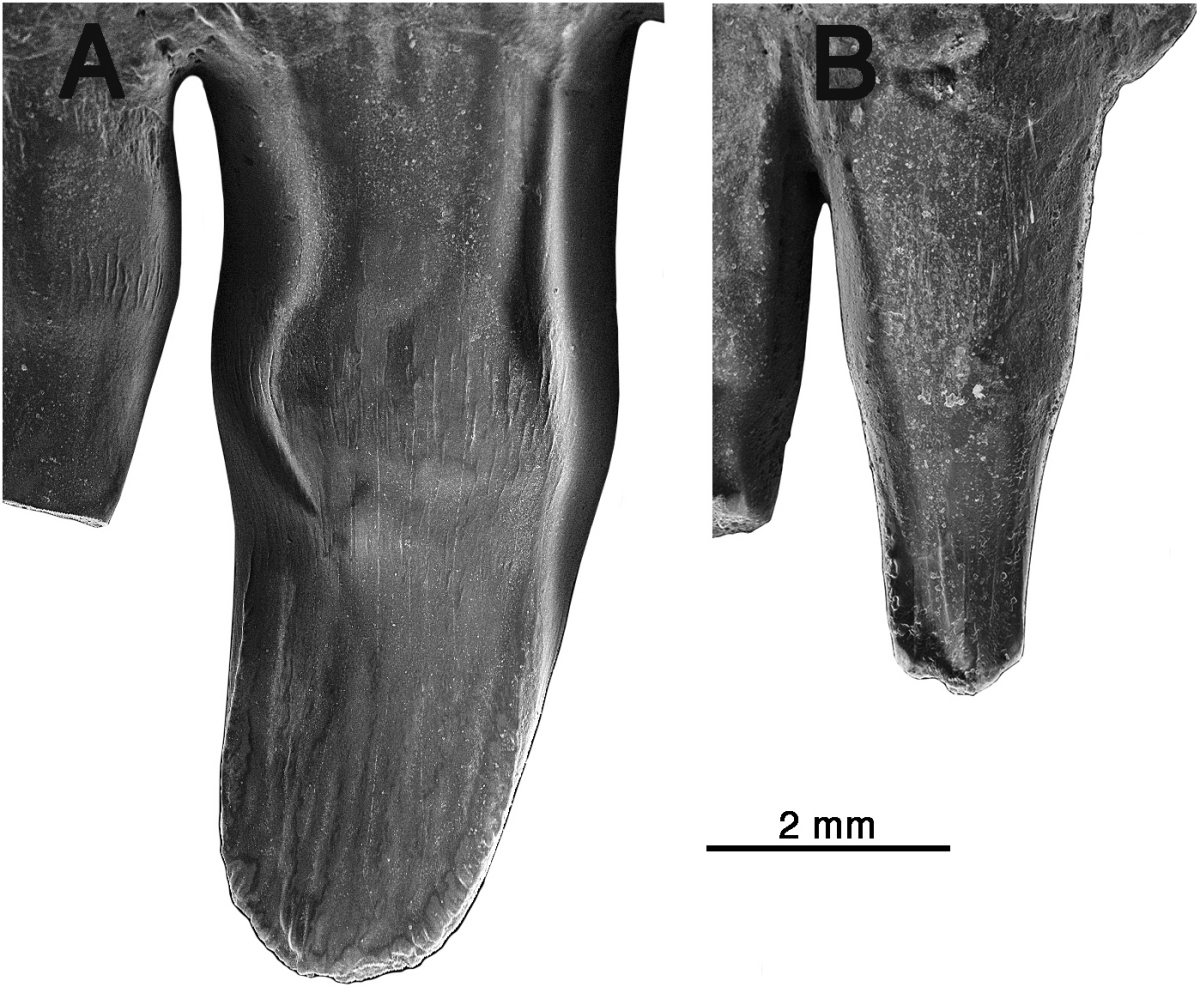
Comparison of premaxillary teeth of *Oromycter* and *Arisierpeton simplex.* (A) SEM image of lingual surface of second premaxillary tooth of *Oromycter* (FMNH PR 2283); (B) SEM image of lingual surface of second premaxillary tooth of *Arisierpeton* (GAA 00239).

**Figure 4:**
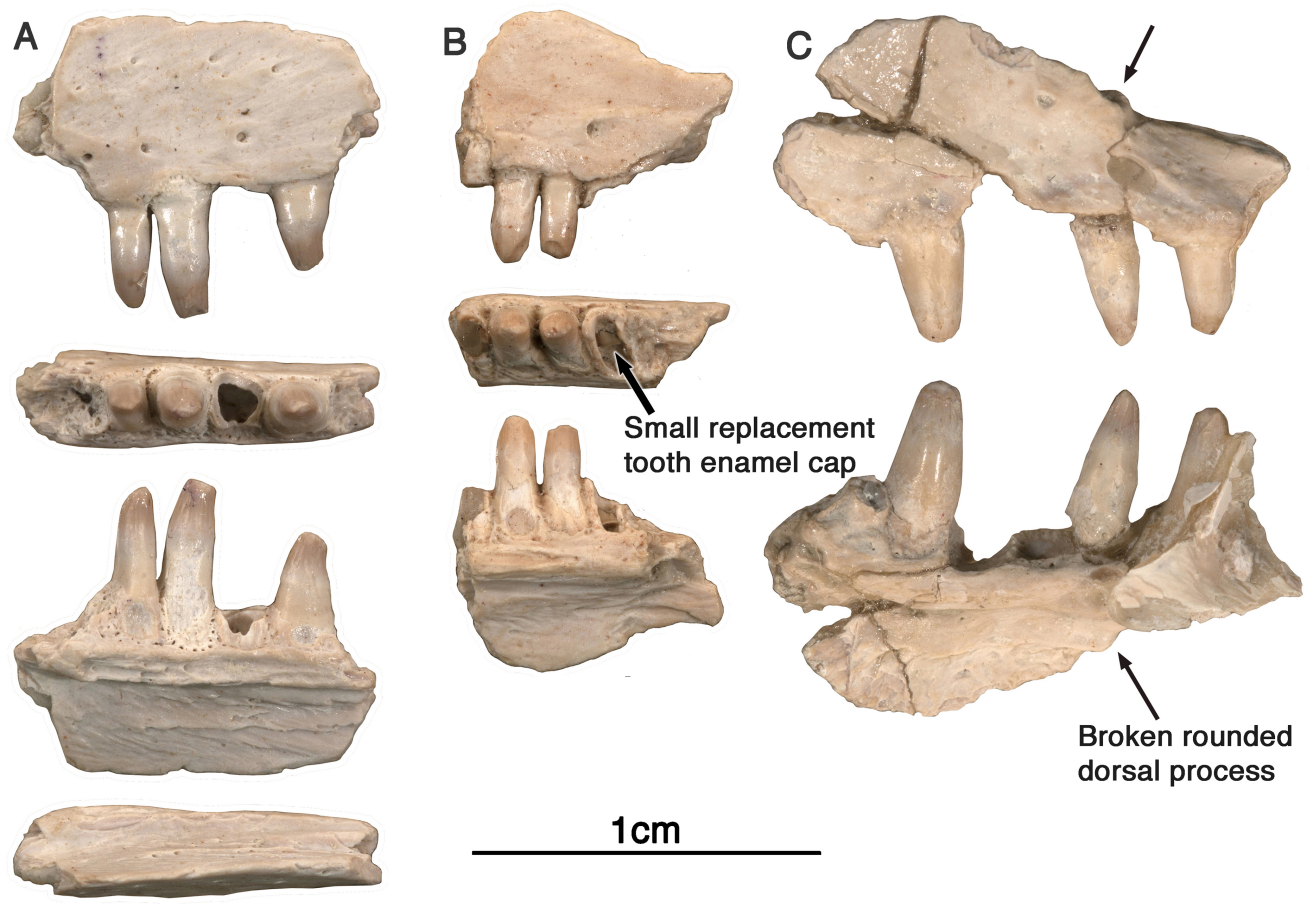
*Arisierpeton simplex* maxillae. (A) Images of left maxillary fragment GAA 00207 in lateral, ventral, lingual, and dorsal views; (B) images of small right maxillary fragment GAA 00240 in lateral, ventral and ligual views; the ventral or occlusal view shows the presence of an unerupted tooth at the base of the brocken tooth crown, as indicated by an arrow; (C) GAA 00225-2, anterior fragment of right maxilla in labial and lingual views. Arrow points to base of rounded anterior dorsal process of maxilla.

**Figure 5:**
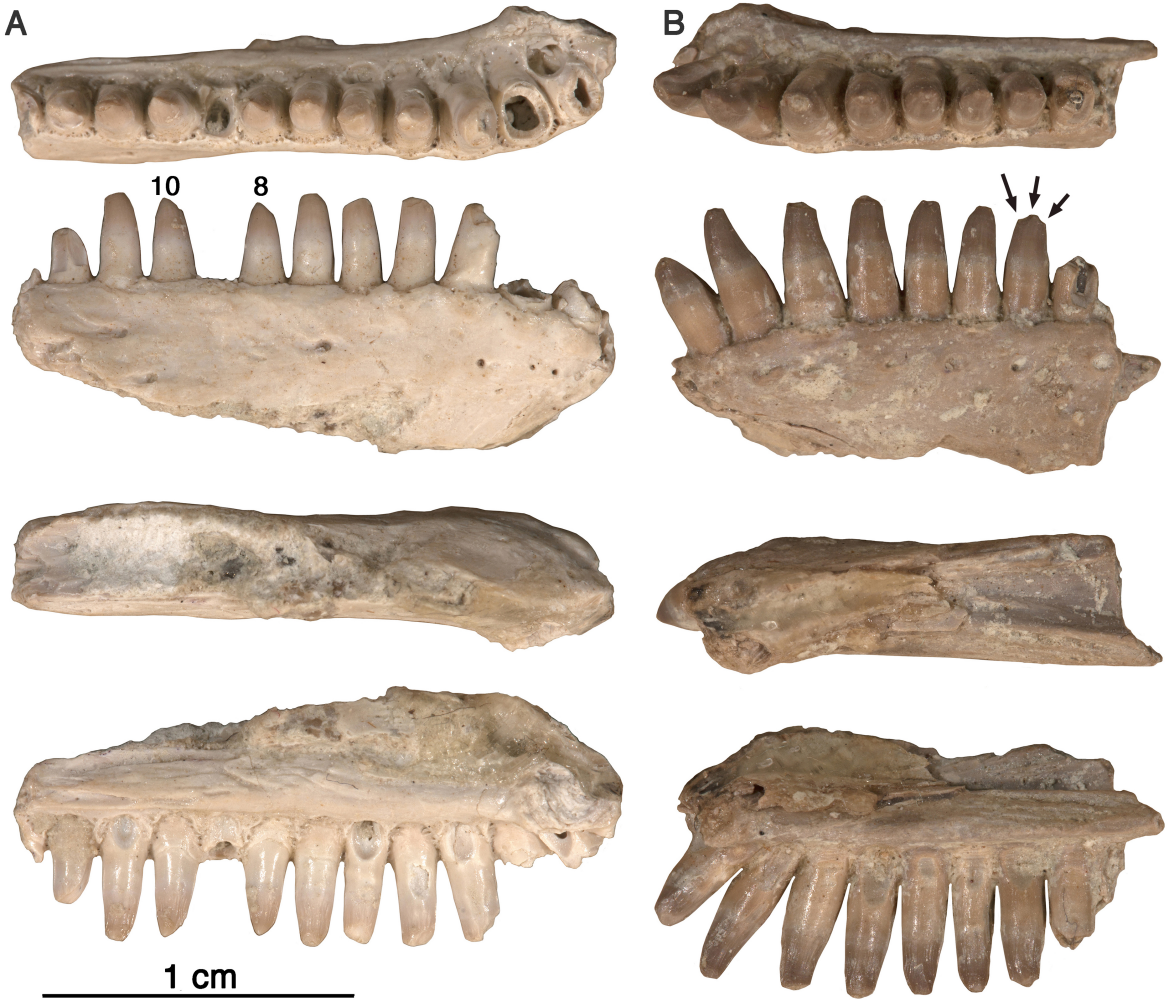
*Arisierpeton simplex* dentaries. (A) Images of left dentary GAA 00246-2 in occlusal, labial, dorsal and lingual views; (B) images of right dentary GAA 00246-1 in occlusal, labial, dorsal, and lingual views. Arrows point to incipient cusps of tooth crown. Numbers refer to position in tooth row.

**Figure 6:**
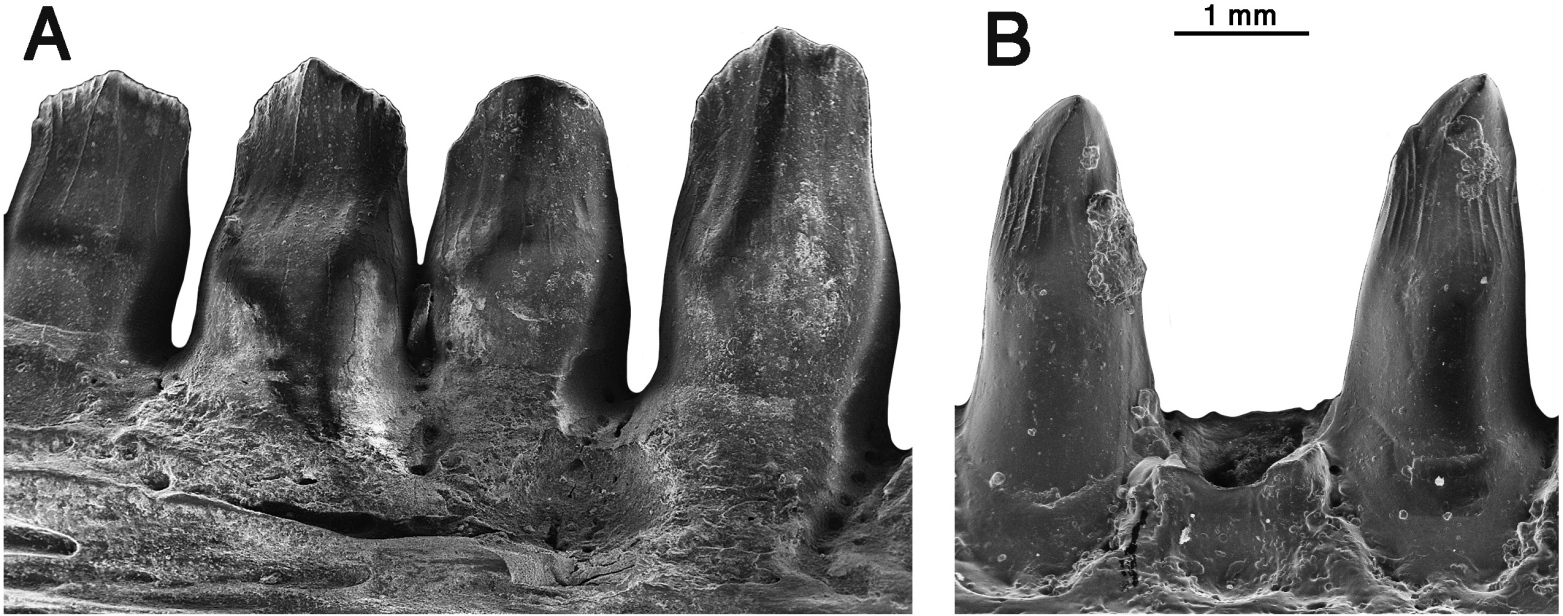
Comparison of dentary teeth of *Oromycter* and *Arisierpeton.* (A) Lingual SEM image of *Oromycter* dentary with four teeth and resorption pit (FMNH PR 2287); (B) lingual SEM image of dentary with two complete teeth (in tooth positions 8 and 10) and a shed tooth position GAA 00246-2.

**Figure 7:**
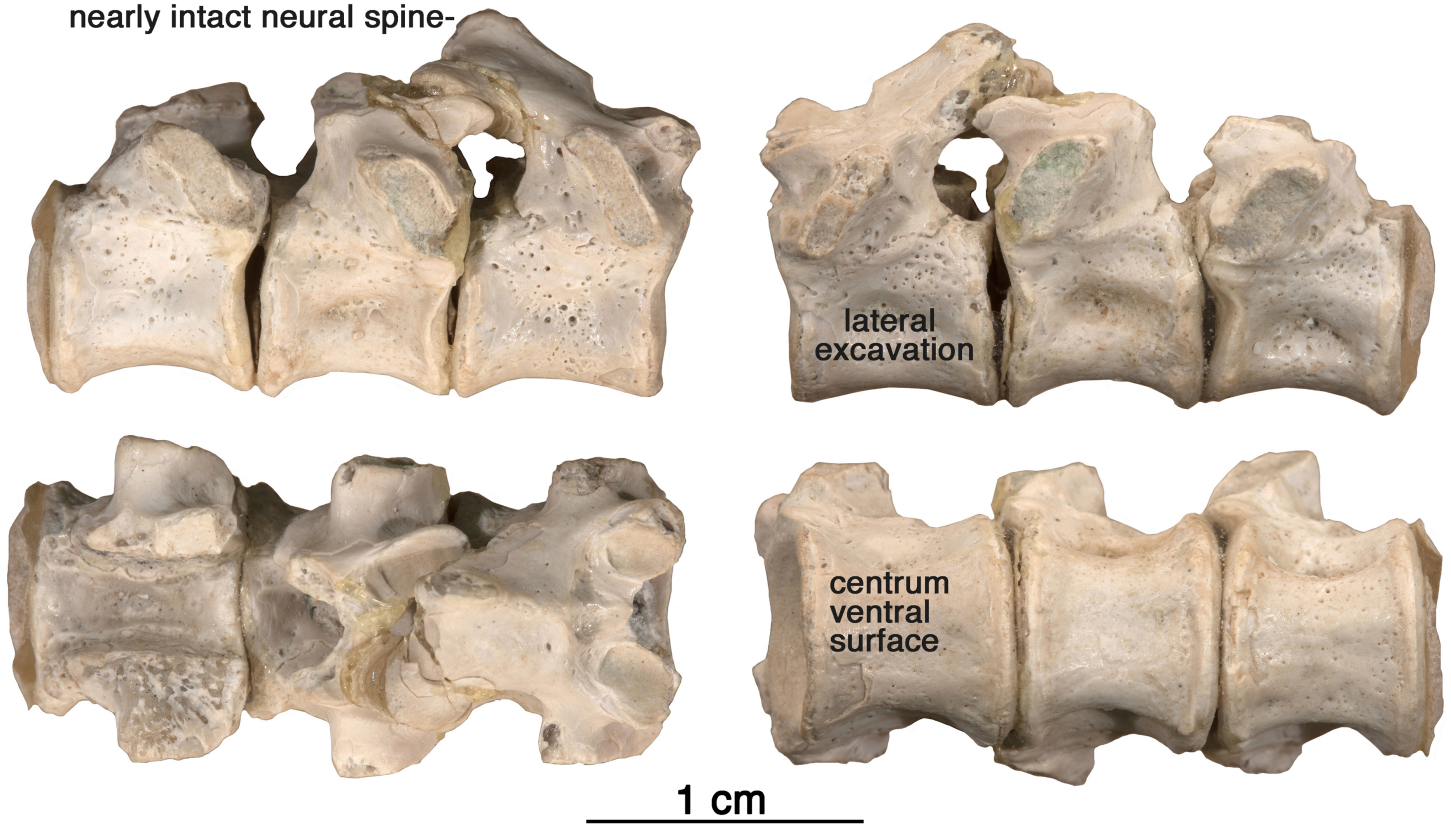
*Arisierpeton simplex* dorsal vertebrae. Images of three posterior dorsal vertebrae, GAA 00244, in right lateral, left lateral, dorsal and ventral views.

**Referred Specimens-**GAA 00242 (Fig. 2A), a right premaxilla; GAA 00239 (Figs. 2B and 3B), a right premaxillary fragment; GAA 00207 (Fig. 4A), a left maxillary fragment; GAA 00225-2 (Fig. 4C), a right maxillary fragment; GAA 00240 (Fig. 4B), a left maxillary fragment with two teeth and fragments of two other teeth; GAA 00246-1 (Fig. 5B), a partial left dentary with eight teeth; GAA 00246-2 (Figs. 5A and 6B), a partial right dentary with 12 teeth or parts of teeth; GAA 00244 (Fig. 7), a series of three dorsal vertebrae.

**Type Locality and Horizon-**Richards Spur, Comanche County, Oklahoma; early Permian (Cisuralian: Artinskian), dated to ca. 289 Ma by Woodhead et al. (2010), making the locality lowermost Artinskian according to the current chronostratigraphic system of the International Commission on Stratigraphy (2018).

**Etymology**-Generic name in honor of Mr. Giuseppe Alberto Arisi, who identified this material and made it available for research. Specific epithet refers to the relatively simpler morphology of the marginal dentition than in other Permian caseids.

**Diagnosis-**Small caseid synapsid characterized by the presence of four premaxillary teeth and by the presence of three modestly developed distal cusps on the premaxillary teeth. As in other caseids the marginal dentition is not recurved, but curved slightly medially. Differs from other caseids with the presence of little or no lingual swelling below the crown. Differs from other caseids in the first premaxillary tooth being sub-equal to the other teeth on this bone. As a monospecific genus, the diagnosis is the same for both the genus and species.

## Description

All specimens show varying levels of damage. The most likely source of the damage is the result of preparation of the specimens by the original collector, but it is also possible that some of the damage is taphonomic in nature. It is clear that the damage to the crowns of the teeth is not the result of tooth wear, as none of the characteristic striations caused by tooth on tooth wear are present. In contrast to the materials of *Oromycter,* the specimens of this caseid are not impregnated by hydrocarbons, and the bones are therefore less well preserved and less resistant to damage. The available evidence suggests that *Arisierpeton* is a caseid, as discussed below, and distinct from the other known caseid from this locality, *Oromycter* (Reisz, 2005)*.* Since they are from the same locality, and their dentitions are well preserved, this description will consider the similarities and differences between these two taxa (see Figs. 3 and 6). The karst deposits near Richards Spur preserve a rich assemblage of terrestrial vertebrates. The vast majority of these taxa and most of the specimens that represent them have strictly carnivorous dentition, and are clearly distinguishable from those of *Arisierpeton* or *Oromycter.* The dentition of four other taxa at Richards Spur are sufficiently different to merit some comparisons with those of the two caseids listed above. The captorhinids *Captorhinus,* and *Opisthodontosaurus* have modified their dentition from the primitive captorhinid pattern by having bulbous crowns. *Captorhinus* is known for its characteristic ogival dentition (LeBlanc & Reisz, 2015), while *Opisthodontosaurus* (Reisz et al., 2015) has a distinctive morphology, with a wide, circular base, and a bulbous crown that tapers to a blunt point. These are therefore clearly quite different from the teeth seen in the two caseids from Richards Spur. Similarly, the parareptile *Bolosaurus* (Reisz, Barkas & Scott, 2002; MacDougall, LeBlanc & Reisz, 2014) has a distinctive marginal dentition that ranges from relatively slender anterior, slightly procumbent teeth that have extensive wear facets on the crown for occlusion with their counterpart, while the cheek teeth have bulbous dentition with characteristic vertical fluting and again large wear surfaces. Finally, Richards Spur also has the remains of diadectid teeth (Reisz & Sutherland, 2001), which not only have extensive wear facets caused by tooth on tooth occlusion, but also have deep roots with plicidentine. Irrespective of the anatomy of the skull elements, the dentition of *Arisierpeton* sets it apart from all other taxa at Richards Spur, but makes comparisons with *Oromycter* informative within the context of caseid evolution.

**Premaxilla**—Three right premaxillae are preserved, GAA00225-1 (Fig. 1), GAA00239 (Fig. 2A), and GAA00242 (Fig. 2B), indicating that the holotype and referred specimens represent a minimum of three individuals. In most respects these premaxillae are similar to that of *Oromycter* (Figs. 2 and 6, Reisz, 2005)*,* and quite distinct from other taxa known from Richards Spur. These similarities include the presence of the massive, thick dorsal or nasal process, the small size of the vomerine or palatal process, broad central portion, and the wide maxillary process with a large dorsally located sutural surface for the anterior process of the maxilla. In typical caseid fashion, the dorsal or nasal process is tilted anteriorly and as in caseids, the anterior surface of the bone is pierced by large foramina (Olson, 1968; Maddin, Sidor & Reisz, 2008). Interestingly, there is also a foramen that opens dorsally at the base of the dorsal process (Fig. 1, arrow), but it is substantially smaller than in *Oromycter.*

In contrast to the condition seen in most caseids and even caseasaurs (the more inclusive clade comprising caseids and eothyridids), there are four premaxillary teeth. Most other caseasaurs have three or two premaxillary teeth, often with the first tooth being the largest tooth in the marginal dentition (Maddin, Sidor & Reisz, 2008; Reisz, Godfrey & Scott, 2009; Brocklehurst et al., 2016). In *Arisierpeton,* the premaxillary teeth are all similar in size. In these two features, number and size of teeth, *Arisierpeton* is readily distinguishable from *Oromycter* and other caseids*.* The similar size of premaxillary teeth is probably not a function of ontogeny, or tooth replacement pattern. This is supported by the dental pattern seen in the ontogenetic series of skulls of the caseid *Ennatosaurus tecton* (Olson, 1968; Maddin, Sidor & Reisz, 2008; R Reisz, pers. obs., 2008), in which the expected heterodonty is retained at all known stages. *Ennatosaurus* is the only known caseid in which we have several well preserved skulls ranging in size from 91 mm in length (PIN 1580/24) to 170 mm in length (PIN 1580/17). *Arisierpeton* resembles the small European caseid *Euromycter* (Reisz et al., 2011) in having four premaxillary teeth, but in that much younger caseid the first premaxillary tooth is larger than the other premaxillary teeth and the largest of the marginal tooth row. This similarity in premaxillary tooth count is a rather surprising finding because *Euromycter* is phylogenetically positioned more deeply nested within Caseidae than either *Oromycter* or *Casea broilii* (Maddin, Sidor & Reisz, 2008; Brocklehurst et al., 2016)*.* Although the presence of four or more premaxillary teeth is primitive for amniotes, caseasaurs including eothryridids (Brocklehurst et al., 2016) are derived in having reduced their premaxillary dentition to three or two teeth. This suggests that the presence of four premaxillary teeth in *Arisierpeton* and reduced heterodonty may be autapomorphies of this taxon.

Most of the premaxillary teeth on all specimens are damaged near their apices but do show clear evidence of tapering crownward. This is most evident in tooth number two and four in the holotype GAA 00225-1 (Fig. 1), with more pronounced tapering labio-lingually than anteroposteriorly, as is expected in other caseids. However, all of the preserved teeth also show that they are somewhat spatulate towards the tip of the crown and are unlikely to have had a pointed apex. Their spatulate condition indicates that the teeth of *Arisierpeton* shares this condition with *Oromycter,* while the more basal *Eocasea* and the eothyridids have simple, conical, slightly recurved teeth (Reisz, Godfrey & Scott, 2009). In addition, in other caseids, the teeth remain spatulate and often have anteroposteriorly expanded crowns, with denticles along the edge of the crown.

It is fortunate that one tooth is complete in the three premaxillae, and this is in the largest, and presumably most mature specimen, GAA 00242 (Fig. 2B). Based on the shape of the preserved portion of the premaxilla, the complete tooth is probably the third tooth in the series. Here, the tip of the crown is complete, and shows that the tooth is tricuspid, with two small accessory cusps that are separated from the central tip of the tooth by ridges that run the height of the crown. Although tooth number three is damaged in the holotype, tooth number two is more intact, and shows a more modest expression of the features seen in the third tooth of the larger premaxilla. This raises the possibility that there may be some ontogenetic variability in the presence of these accessory cusps, as evidenced by the differences between the smallest and largest premaxillae, with the middle-sized holotype specimen showing an intermediate condition. The best preserved premaxillary tooth of *Oromycter* is the second tooth in the series and can be compared with that of *Arisierpeton.* As seen in Fig. 3A, it is broadly spatulate. Although this tooth has external and internal vertical fluting on the crown, the tooth is broad at the tip, without any accessory cusps.

**Maxilla—**Three maxillary fragments (Fig. 4) have been recognized, GAA 00207 (left maxillary fragment from the posterior region with three teeth and one root fragment), GAA 00225-2 (right maxillary fragment from the anterior region with three preserved teeth, and two root fragments), and GAA 00240 (right maxillary fragment with two full teeth, two root fragments, and one unerupted tooth crown). Although all of the maxillae are quite fragmentary, they do show some morphological features, as described below, that allow us to identify them as all belonging to this taxon, and to evaluate them. They also provide some valuable anatomical information about the dentition of this cranial element.

The available evidence suggests that the maxillary dorsal process, similar in shape to other caseids (Maddin, Sidor & Reisz, 2008), may have been slenderly built, and similar in some respects to the observed anatomy in *Oromycter.* There appears to be a modest anterodorsal process of the maxilla, as seen in GAA 00240 (Fig. 4C), at the level of the internal narial border of the bone medially. Its dorsal terminus is broken, making it difficult to determine its original height. However, the most remarkable feature of this maxillary fragment is the morphology of the anterior edge of the anterodorsal process. In *Oromycter* there is no anterior maxillary notch for the anteriorly facing shelf that is associated with the greatly enlarged external naris of all other caseids. In contrast to *Oromycter,* in *Arisierpeton* the preserved base of the narial border of the dorsal process is wide and rounded, suggesting that an anterior maxillary shelf may have been present on the complete maxilla (Fig. 4C, arrow). It is not possible to determine with certainty if this shelf was present in *Arisierpeton,* but the shape of the maxilla in this region does suggest that there may have been one. Even if a fully developed shelf was not present in *Arisierpeton,* the morphology of the maxilla represents a more derived condition than that seen in *Oromycter* (Reisz, 2006, Figs. 1C and 3A)*,* where the anterior edge of the dorsal process is a sharp ridge, and the distinctive anterolateral narial shelf is restricted to the lacrimal bone. On the anterior lateral surface of the maxilla, instead of a series of relatively large labial foramina situated along the external surface of the bone, only a single large anteriorly oriented foramen is present in *Arisierpeton* (Figs. 4B and 4C)*.* External surface sculpturing is also more modest than in *Oromycter* and is restricted to faint grooves associated with small foramina on the surface of the bone.

Most of the maxillary teeth are damaged in some way, with the exception of the second tooth on GAA00225-2 (Fig. 4C), and the most anterior tooth of GAA 00207 (Fig. 4A). Unfortunately, the preserved maxillary tooth rows are too short to determine if heterodonty was present. All the teeth show the typical caseid morphology of having lingually curved crowns, with little or no recurvature. Of particular interest is the un-erupted tooth preserved in the partially resorbed tooth in GAA 00240 (Fig. 4B, arrow, occlusal view). As is typical of teeth in their early stages of development, prior to implantation, only the enamel cap is preserved, a simple conical structure. Although difficult to discern, a small secondary cusp is present along the posterior edge of the cap. The tooth immediately anterior to this un-erupted tooth has a similar superficial morphology as those on the premaxilla, with vertical fluting lingually on the enamel surface. Unfortunately, most of the teeth have lost their crown tips, making it difficult to determine if they were also tricuspid. However, the arrangement of the fluting suggests that there also was a central cone in these teeth and possibly two accessory cusps, or at least incipient accessory cusps (Fig. 4B, lingual view).

**Dentary—**Two anterior dentary fragments are preserved, GAA 00246-2 (Fig. 5A), a right dentary with seven complete or near complete teeth and 5 tooth fragments, and a left dentary GAA 00246-1 (Fig. 5B), with eight teeth, and a single small anterior tooth fragment. Assignment of these dentaries to a caseid is based on the shape of the symphyseal area. As in other caseids, the dorsal edge of the dentary bone curves ventrally near the symphysis, and forms an acute angle with the ventral edge of the bone. This results in a substantially more slender dentary bone near the symphysis than in the rest of the bone. As in other caseids, this is related to the presence of a well-developed anterior process of the splenial bone, one that contributes to a large portion of the symphysis medially. Although the dentary bones are nearly complete anteriorly, their morphology cannot confirm the entire depth or height of the lower jaw at the symphysis. This is because the dentary contributes only to the dorsal half of the symphysis in caseids, and the splenial most likely contributed to the symphysis, as it formed the lower part of the symphyseal region of the mandible. The assignment of these dentaries to this small caseid is based mainly on dental features, and to some extent on the labial surface characteristics of the bone. The teeth of these dentaries are identical to those found on the maxillae, as discussed below. In addition, the surface characteristics of the labial side of the dentaries are similar to those of the maxillae, showing little sculpturing, and occasional small foramina. A well-developed, anteriorly extending Meckelian canal is formed by the dentary bone, below which it would be suturally attached to the splenial.

As in all caseids, the anterior teeth of the dentary lean forward. In both dentaries, the root or fragment of a very small anterior tooth is preserved. The anteriorly tilting first three teeth are the largest of the dentary tooth series, based on their perceived height (when preserved) or the diameter of the root portion of the teeth (when broken). This is consistent with the condition in most caseids, including *Oromycter, Cotylorhynchus,* and *Casea broilii* (Olson, 1968; Reisz, 1986)*.*

Despite the presence of numerous dentary teeth in these two specimens, only in GAA 00246-2 (Fig. 5A) are tooth positions 8 and 10 intact at the tip of the crown. The intact teeth in GAA 00246-2 (Fig. 6B) are smaller than teeth anterior and posterior to them, and they carry the same kind of vertical fluting lingually as seen in the upper teeth. The apex of each tooth carries anterior and posterior carinae, with a slight hint of an accessory cusp associated with the fanning of the anterior fluting from the central cone of tooth. There is no evidence of a posterior cusp where the fluting extends to the posterior carina. These teeth also appear to have a slightly posteriorly tilted central cone, but in typical caseid fashion there is clear evidence of a pronounced lingual tilt to the crown. As far as can be determined, all of the dentary teeth conform to this pattern, although one tooth at tooth position 8 in GAA 00246-1 (Fig. 5B), although slightly damaged at the tip, does appear to have both anterior and posterior cusps. In all cases the teeth are more slender than in the dentaries of *Oromycter* and have more modest lingual shoulders at the base of the crown.

Overall, it appears that the dentition in *Arisierpeton* shows some modifications in tooth shape and crown outline from the primitive amniote condition seen in the basal caseid *Eocasea* and in eothyridid caseasaurians. The teeth show little or no recurvature, but instead have some medial or lingual curvature apically. The crowns, when preserved, show some fluting and occasional carinae, which are sometimes sufficiently well developed for the formation of a tricuspid terminus, somewhat reminiscent of the condition seen in *Cotylorhynchus* (Maddin, Sidor & Reisz, 2008)*.* However, the kind of bulging of the lingual side of the tooth below the crown seen in *Cotylorhynchus,* and geologically younger caseids is only modestly developed in *Arisierpeton.*

**Vertebrae—**The string of three posterior dorsal vertebrae GAA 00244 (Fig. 7), were found in the same pocket as the dentigerous elements, but their association with the holotype and other referred specimens is tentative. The vertebrae have the typical cylindrical, spool-shaped centra, with shallow lateral excavations, but the most striking feature of the centra is the presence of flat ventral surfaces between the rounded anterior and posterior articular surfaces, as seen in the well preserved vertebrae of *Ruthenosaurus* (Reisz et al., 2011). The centra are solidly fused to the neural arches, which carry well-developed, massive, transverse processes, short zygapophyseal surfaces, and slender, presumably anteroposteriorly short, simple neural spines (Romano et al., 2017). The transverse processes are short and stout, and were not fused to the ribs. The size of the transverse processes indicate that these are most likely posterior dorsal vertebrae (Stovall, Price & Romer, 1966). A pair of small excavations are present dorsally on the neural arch, between and slightly posterior to the prezygapophyses. There is no evidence of ventral excavation of the centra for intercentra, a common feature of caseid posterior dorsal vertebrae. Thus, the morphology of the centra and neural arches are entirely in agreement with known caseid morphology (Reisz et al., 2011). They can be assigned with confidence to a small caseid. However, their assignment to *Arisierpeton* is tentative and based on co-occurrence and size, and there are no recognizable diagnostic features on the centra below the family level.

## Discussion

Although restricted to three cranial elements, the premaxilla, maxilla, and dentary, the anatomy of these dentigerous elements is sufficiently different from that of other taxa found in this upland locality (MacDougall et al., 2017) to erect a new taxon. The assignment of these dentigerous elements and the vertebrae to caseids is based on a suite of characters that distinguish that clade. Notable among these are the overall morphology of the premaxilla, maxilla, and dentary and the patterns of foramina on their labial surfaces. In addition, the forward tilting of the premaxilla and the procumbent dentary teeth and the absence of an enlarged “caniniform tooth” are clear caseid features. The morphology of the teeth, especially the crowns with their lingual curvature are similar to other caseids and are distinct from other amniotes. From a narrower taxonomic perspective, similarities between the teeth present on the dentigerous elements, the labial surfaces of the bones and the pattern of distribution of foramina, all provide strong evidence that these elements belong to a single caseid taxon, *Arisierpeton.* The vertebrae, although clearly caseid in morphology, cannot be assigned with confidence to this taxon, and its identification remains tentative.

In the broader context of caseid dental evolution, the primitive condition is represented by the presumed carnivorous basal caseid *Eocasea* and the eothyridids, all having relatively simple conical, slightly recurved teeth with pointed apices (Reisz & Fröbisch, 2014; Brocklehurst et al., 2016). Both caseids from Richards Spur show the derived conditions of lingual curvature, a spatulate crown, and some level of lingual bulging at the base of the crown. The dentition of *Oromycter* has the crown morphology mentioned above but there are no cusps along the edge of the crown. Nevertheless, it is reasonable to suggest that the spatulate tip of the crown could be used to crop plant materials (Figs. 3A and 6A). All other caseids more deeply nested within Caseidae appear to have evolved some additional cusps at the top of the crown, as seen in its most primitive condition in *Arisierpeton.* Cusp morphology varies significantly among caseids, and apical dental complexity does not appear to be related to size or to caseid tree topology (Maddin, Sidor & Reisz, 2008). Recent phylogenetic analyses (Brocklehurst et al., 2016; Reisz & Fröbisch, 2014) have confirmed the general tree topology of caseid synapsids, with *Eocasea* as the sister to all other members of the clade. The other caseid taxa that have well preserved dentition, are positioned with *Oromycter, Casea broili, Euromycter, Ennatosaurus* sequentially more deeply nested within Caseidae, and with *Angelosaurus* and *Cotylorhynchus* as sister taxa (Reisz & Fröbisch, 2014). *Oromycter* teeth are spatulate but do not have any cusps, *Casea* and *Cotylorhynchus* have three cusps, while *Euromycter, Ennatosaurus,* and *Angelosaurus* have five or more cusps, indicating that either convergence or reversal of dental complexity was a factor in caseid evolution. It is for this reason that I do not think that dental morphology alone can inform us about the exact position of *Arisierpeton* within caseid evolution.

Historically, the Dolese Brothers Limestone Quarry at Richards Spur has yielded tens of thousands of bones and occasional partial skeletons that have been impregnated with hydrocarbons from the underlying oil fields of the Woodford Shale (Donovan et al., 1992; MacDougall et al., 2017). Consequently, these bones are dark brown to black in color and are very easy to see within the cave sediments. The specimens that are embedded in clay-rich sediments are easily removed by the use of dilute acetic acid and are predominantly disarticulated in nature, with only rare occurrences of articulated skulls or skeletons being present. The most common elements of the Dolese cave system community preserved in this manner belong to the small amphibamid *Doleserpeton* (Sigurdsen & Bolt, 2010; MacDougall et al., 2017) and the small captorhinid eureptile *Captorhinus aguti* (Heaton & Reisz, 1980). These types of fossil remains have been collected during the last nine decades. Of particular relevance here is the discovery of fragmentary material of the basal caseid *Oromycter* (Reisz, 2005), in this kind of sediment. *Oromycter* was collected by Dr. John Bolt before the end of the last century, but was not recognized as a distinct taxon at the time. Recently exposed caves in the quarry (2005) have yielded abundant remains of dissorophoid temnospondyls, including several semiarticulated and articulated specimens in calcite-rich sediments (Gee & Reisz, 2018). These new materials could not be prepared in the traditional fashion of acidic dissolution, and required mechanical preparation. These recently exposed caves also yielded the remains of a new varanopid synapsid and a sphenacodontid (Maddin, Evans & Reisz, 2006; Evans, Maddin & Reisz, 2009), increasing the taxonomic diversity of synapsids to four at the Richards Spur locality, but synapsids remain rare elements of the overall vertebrate community. This is in strong contrast to the diversity of dissorophoid temnospondyls (seven taxa), parareptiles (eight taxa), and eureptiles (eight taxa) that have been discovered at this locality (MacDougall et al., 2017).

An unusual, large cave deposit was exposed in the last decade of the 20th century, one that lacked the typical hydrocarbon impregnation seen in the other parts of the cave system in the quarry. Thus, in contrast to the vast majority of dark brown or black colored fossil materials (“black bone”) derived from Richards Spur, these fossils were cream-colored, almost white in colour (“white bone”). The fauna preserved in this cave system includes numerous disarticulated materials of the trematopid *Acheloma* (Sullivan & Reisz, 2000)*,* and the large captorhinid *Captorhinus magnus* (Kissel, Dilkes & Reisz, 2002). The specimens of *Arisierpeton* were derived from this distinctly colored assemblage of white bones. Despite the differences in the composition of the most abundant taxa, there are similarities between the two types of communities, with the dissorophid *Cacops* (Reisz, Schoch & Anderson, 2009) and the captorhinid *Captorhinus aguti* also being present in both assemblages, but being rarer in the “white bone” assemblage. The one major difference is the apparent rarity of the small amphibamid *Doleserpeton* in the “white bone” assemblage. Given the similarity in colour between the matrix and the bones, the recognition of the tiny skeletal elements of this small amphibamid is very difficult. In addition, the white bones appear to be softer than the black bone, and separation between the bone and the surrounding matrix is poor. It is likely that the standard procedure of screen washing that was used in separating the bones from the soft matrix of the “black bone” assemblages did not work well for the softer “white bone” assemblages. In addition, the collection of the “white bone” assemblage was done by two commercial collectors who focused on the larger materials, and who may have missed or ignored the smaller bones.

The caseid *Oromycter* is known from the “black bone” community, while the new caseid *Arisierpeton* is known from the “white bone” community. Currently, there is no clear understanding of the taphonomic and paleoecological implications of these two communities with apparently different relative abundances, but differences in taxic diversity of the captorhinids (LeBlanc et al., 2015) raises the possibility that we are looking at community evolution in the region, and that different parts of the cave system may preserve different time slices of the community that lived in the area around the caves. Unfortunately, there is no stratigraphic control available at the Dolese Brothers Limestone Quarry, in part due to restricted access to the quarry during daily operations. However, it is possible that the spatial separation between the various cave chambers that preserved the fossils may have resulted not only in differences in relative abundances of taxa, but also in some differences in the taxonomic composition of the communities that lived in this region during the early Permian.

There are two caseids preserved in the large Dolese Brothers Limestone Quarry at the Richards Spur locality, but these were collected from two cave infills that were probably spatially separated by at least 300 m. It is not possible to determine if they were coeval in age or separated stratigraphically or temporally. Although the available materials are too fragmentary for a phylogenetic analysis, both taxa appear to be basal caseids. The high premaxillary tooth count of *Arisierpeton* can be interpreted either as being autapomorphic for this caseid, given that other caseids have fewer teeth and the immediate eothyridid outgroup possesses fewer teeth as well, or as a reversal to the primitive condition of 4–5 premaxillary teeth widely found among early amniotes. The modest level of bulging of the body of the tooth appears to represent the primitive condition, and is significantly less developed than in *Oromycter* or other caseids*.* Finally, the presence of incipient tricuspid crowns, and the possible presence of an anterior emargination of the dorsal process of the maxilla, may also suggest that in these features *Arisierpeton* is somewhat more derived than *Oromycter,* but still more basal than other caseids. It is notable that all the known skull elements, including the premaxillae, maxillae, and dentaries of *Arisierpeton* are smaller than the known skull elements of *Oromycter,* and their surface sculpture patterns and foramina are more modestly developed than in the latter taxon. However, the known anatomical differences preclude the possibility that they represent different ontogenetic stages of the same taxon (Maddin, Sidor & Reisz, 2008).

In most caseids from younger strata in Laurasia, the ratio between skull and the trunk is unusually small, with the largest taxa having diminutive skulls relative to the size of their overall skeleton. It is presumed that the relatively small size of the skull in these large bodied synapsids is related to their high fiber herbivory. The oldest known caseid, the small late Carboniferous *Eocasea* (Reisz & Fröbisch, 2014)*,* has a skull/trunk ratio that is similar to other faunivorous amniotes, and does not appear to have any of the morphological or dental features that are associated with herbivory. A recently discovered skull fragment of the holotype skull (KUVP 9616) has preserved the dentigerous region, and appears that the maxilla may be equal in length to nearly 4 dorsal vertebrae. However, the holotype and only known specimen is a juvenile, and its head/trunk ratio may have been affected by this early ontogenetic stage.

It is not possible to determine if the presumed herbivorous dentition of *Arisierpeton* is associated with any other morphological features that generally reflect herbivory in this clade of basal synapsids. If we assume that most of these skull elements are of a similar ontogenetic stage with the vertebrae (and are the same taxon), then the skull-to-trunk ratio in *Arisierpeton* could represent the primitive condition for caseids (skull length = 6.5 dorsal vertebrae), and similar to that in the eothyridid *Vaughnictis* (Brocklehurst et al., 2016). Younger, larger caseids are characterized by having very small skull to trunk ratios, most spectacular among these being *Cotylorhynchus* (skull length = 3.4 dorsal vertebrae. R Reisz, pers. obs., 2016). However, given the fragmentary nature of the *Arisierpeton* materials, and the tentative nature of the assignment of the vertebrae, only future discoveries of caseid materials at this locality will allow us to shed more light into the early stages of the evolution of herbivory in this clade.

## Conclusions

The fossiliferous cave deposits of Richards Spur preserve a highly diverse assemblage of terrestrial vertebrates. Although synapsids are represented in this community, they are relatively rare, with only a handful of taxa being present. Of these, the new caseid taxon *Arisierpeton,* is represented by readily recognizable tooth bearing elements and possibly by three vertebrae. The assignment of *Arisierpeton* to caseids is based on the morphology of the dentigerous elements, while the assignment of the vertebrae to this taxon is tentative and based on their preservation in close association with the dentigerous elements, and by distance from that of *Oromycter*. However, the distinctive ventral surface of the centra makes their assignment to caseids well founded. Like the other four synapsids at this locality, *Arisierpeton* appears to be a rare component of the community dominated by eureptiles and dissorophoid temnospondyl amphibians. Future work may uncover other taxa of synapsids, but the overall community structure is likely to stay unchanged.

## Institutional abbreviations

FMNH: Field Museum of Natural History, Chicago, USA
GAA: Giuseppe Alberto Arisi Collection, Minister a la activita Cultural, Italy
KUVP: University of Kansas Natural History Museum, Lawrence, Kansas, USA
PIN: Palaeontological Institute, Russian Academy of Science, Moscow, Russia

## Anatomical abbreviations

dp: dorsal process of premaxilla
ms: medial survade of premaxilla
mx: sutural surface on maxillary process of premaxilla

## Additional Information and Declarations

### Data Availability

The following information was supplied regarding data availability: The fossil materials studied here belong to the Arisi Permian collection (DPCR 022/17 dated 19 April 2017) that is legally registered by a decree of the Ministero per i Beni e la Attivita Culturali. The Spritendenza per i Beni Archeologici has authorized the author to study this collection.

### New Species Registration

The following information was supplied regarding the registration of a newly described species: Publication LSID: urn:lsid:zoobank.org:pub:B97895AC-87A0-407E-9761-026B71899524 *Arisierpeton* LSID: urn:lsid:zoobank.org:act:42D63CEE-144E-4593-A448- 3B7AEA2D8D69 *Arisierpeton simplex* LSID: urn:lsid:zoobank.org:act:AD7D8FF9-6269-4B1C-A4DD-6F42B969F167.
